# Population Numbers and Physiological Response of an Invasive and Native Thrip Species Following Repeated Exposure to Imidacloprid

**DOI:** 10.3389/fphys.2020.00216

**Published:** 2020-03-27

**Authors:** Xiaoming Zhang, Ru Li, Changxiong Hu, Guohua Chen, Haiyun Xu, Zhixing Chen, Zhengyue Li

**Affiliations:** ^1^National Key Laboratory for Conservation and Utilization of Biological Resources in Yunnan, College of Plant Protection, Yunnan Agricultural University, Kunming, China; ^2^The Key Laboratory of Integrated Pest Management on Crops in South China, Ministry of Agriculture and Rural Affairs, Guangzhou, China; ^3^College of Life Sciences, Hebei University, Baoding, China; ^4^Kunming Hongzhihua Horticulture Co., Ltd., Kunming, China

**Keywords:** imidacloprid, *Frankliniella occidentalis*, *Frankliniella intonsa*, physiological enzyme, interspecific competition

## Abstract

*Frankliniella occidentalis* and *F. intonsa* are devastating pest insects that target *Rosa rugosa*, *Chrysanthemum morifolium*, and *Phaseolus vulgaris*, which are important economical horticultural plants in China. Meanwhile, *R. rugosa* and *C. morifolium* are important cash plants in Kunming, South China. We focus on the population performance of these two thrips species on these three host plants with or without repeated exposure to imidacloprid in Kunming. In the field, the population numbers of *F. occidentalis* developed faster and were larger on these three sampled host plants, especially under imidacloprid exposure, compared with *F. intonsa*. The activity of the detoxifying enzymes (CarE, AchE, and MFO) and the antioxidant enzymes (CAT and POD) in both thrips species were significantly enhanced under imidacloprid exposure, whereas the activities of SOD in both thrips were significantly decreased on these three host plants, compared with the control. Overall, enzyme activity of *F. occidentalis* showed a greater increase than that observed in *F. intonsa* in most cases, which could be exploited in further studies on thrips resistance management.

## Introduction

The western flower thrips [*Frankliniella occidentalis* (Pergande)] (Thysanoptera: Thripidae) was first reported in 1895 in California, United States. It is an invasive species and range expansion has meant that it is the dominant thrip species in most areas in which it has invaded (Morse and Hoddle, 2006; Baez et al., 2011). *F. occidentalis* was accidently introduced into Yunnan province in China in 2000; the species has spread rapidly to other areas of China and has caused serious economic loss in several provinces (Lu et al., 2011). The species often coexists with *F. intonsa* (Trybom) in many agriculture areas. *F. intonsa* is a native species, widely distributed in the Yunnan province, and causes substantial damage to many horticultural and crop plants, such as flowers, lentils, sugarcane, vegetable, and rice. Its nymph and adult stages gather and feed in the flower, petals, and floral organs causing white strip, which then changes to black and brown strip after sunlight exposure. Flowers also show premature senescence when *F. intonsa* populations reach high numbers on hosts (Han et al., 2015). Both thrips species share many vegetables, fruit trees, flowers, and other crop hosts, as well as feed on the flowers and young parts of plants. At present, the control of *F. occidentalis* and *F. intonsa* mainly depends on chemical control, and the use of a large number of chemical insecticides has led to the rapid rise of resistant populations and the reemergence of *F. occidentalis* population (Chen et al., 2011; Zhang et al., 2017b).

Imidacloprid is a neonicotinoid insecticide widely used as a foliar application in horticultural crops to control thrips in China (Wang et al., 2016; Zhang et al., 2017b). Its insecticidal mechanism is to simulate acetylcholine and can be accepted by the acetylcholine receptors of insects. Imidacloprid cannot be decomposed by the acetylcho-linesterase (AchE) in the insect nervous system, and therefore can interfere in the normal nervous activities of insects. Insects eventually die due to long-term excessive excitability (Liu and Casida, 1993). The location of acetylcholine in imidacloprid is different from that of pesticides pyrethroids and organophosphorus, so it has different and more efficient effects than the two above-mentioned insecticides. Therefore, it is not easy for target pest species to develop cross-resistance to other insecticides (Abbas et al., 2015), and it has been used to control a broad range of agricultural pests on many crops (Morse and Hoddle, 2006).

Carboxylesterase (CarE), microsomal mixed-function oxidases (MFO), and acetylcho-linesterase (AchE) are three important detoxification enzymes found in insects. Detoxification enzymes play an important role in the decomposition of exogenous toxins, maintain normal physiological metabolism in insects, and are an important mechanism that enable insects to adapt to environmental stress caused by insecticides (Zhang et al., 2017b), extreme temperature (Jeffs and Leather, 2014), and CO_2_ (Liu et al., 2017), etc. For instance, AchE can degrade the neurotransmitter acetylcholine into compounds of choline and an acetate (Soreq and Seidman, 2001). As an important detoxifying enzyme, CarE has been implicated in stress reaction with its relevant metabolic functions, such as catalyzing hydrolysis of ester, sulfate, and amide (Furihata et al., 2004). Major antioxidative enzymes in insects, including superoxide dismutase (SOD), catalase (CAT), and peroxidases (POD), are reported to be involved in insect defense systems (Zhang et al., 2015; Li et al., 2017). Superoxide anion (O_2_^–^) can be converted into oxygen (O_2_) and hydrogen peroxide (H_2_O_2_) by SOD, then H_2_O_2_ can be broken down into oxygen and water by CAT and POD, which protect insects from oxidative damage (Covarrubias et al., 2008; Li et al., 2017). These three antioxidant enzymes are widely distributed in an insect’s body and are sensitive to changes in external environment (Wang et al., 2016; Liu et al., 2017). Research has shown that insecticides have different effects on the insect defense enzyme system, and the changes in the activity of the enzymes are related to the toxic death of insects or related to the development of insect resistance. For instance, lambda-cyhalothrin and imidacloprid can inhibit the activities of both detoxification CarE, AchE, MFO and antioxidant SOD, CAT, POD enzymes secreted by *Clostera anastomosis* L. (Lepidoptera: Notodontidae) (Liu et al., 2014), while the activity of the antioxidant enzymes of *Blattella germanica* (L.) (Orthoptera, Blattellidae) can be enhanced after cyhalothrin stress (Ma et al., 2009). Wang et al. (2016) showed that the use of the insecticides imidacloprid, diflubenzuron, and abamectin at low concentration could promote the activity of antioxidant enzymes (SOD and CAT) and detoxifying enzymes (CarE and glutathione *S*-transferase, GST) in *Ambrostoma quadriimpressum* Motschulsky (Coleoptera: Chrysomelidae). Similarly, Sun et al. (2015) showed that the activities of CAT, POD, and SOD in the *Coloana cinerea* Dworakowska (Homoptera: Cicadellidae) could be enhanced after exposure to sub-lethal doses of avermectin, imidacloprid, and isoprocarb-buprofezin. Zhang et al. (2017a) found that the sensitivity in the *Sogatella furcifera* Horvath (Hemiptera: Delphacidae) population increased through exposure to triazophos, imidacloprid, and pymetrozine. There are some reports related to the interaction among insect, different host plants, and environmental changes. Liu et al. (2011) and Zeng et al. (2008) studies showed that both detoxification and antioxidant enzymes changed significantly after feeding on different host plants for 3 months. A study by Jiang et al. (2017) showed that significant changes were found in the digestive enzyme activity of *F. occidentalis* and *F. intonsa* by using four kinds of host plants to rear these two pest insects.

The flower industry is a growing industry in China, and it has the potential to provide billions of dollars in annual revenue. Because of their high ornamental and landscape values, rose *Rosa rugosa* L. and chrysanthemum *Chrysanthemum morifolium* Ramat are both economically important horticultural and edible plants in the flower industry and, therefore, are widely planted. Kidney bean *Phaseolus vulgaris* L. is the main vegetable grown but is also the major host plant for *F. occidentalis* in China (Liu et al., 2017; Zhang et al., 2017c). The city of Kunming, located in Yunnan Province, China, is the main cultivated area for China’s edible and fresh cut rose, and is the largest area of chrysanthemum seedling production in the world as well (Liu, 2017; Zhang et al., 2017c). These two plant species are usually planted in the same planting base, kidney beans are used to grow around these two flowers in the field under shared nutritional conditions.

It has been speculated that repeated use of insecticides could seriously affect the biological life cycle of *F. occidentalis* and *F. intonsa*, and the net reproductive rate (*R*_0_), the intrinsic rate of increase (*r*_m_), and the growth rate (λ) of *F. occidentalis* were significantly higher than that of *F. intonsa* (Hu et al., 2018). However, it was not fully understood that the mechanism of these two thrips species had different population dynamics on these three plants. Our aims are to elucidate the mechanism of *F. occidentalis* and *F. intonsa* population dynamics on above-mentioned plants under insecticide treatment, and to try to provide early warning for the occurrence of these two thrips in the current and future pesticide application, and also to provide a recommendation for resistance management of both *F. occidentalis* and *F. intonsa*.

## Materials and Methods

### Materials

Reagents and instruments: Physostigmine, and the antioxidant enzyme SOD, CAT, and POD kits were provided by Nanjing Jiancheng Bioengineering Company. Imidacloprid (a.i. rate is 70%, water dispersible granule) was supplied by Jiangsu Kesheng Group Co. Ltd., China.

### Population Counts of Thrips in the Open Fields

From 2014 to 2017, we investigated the population dynamics of *F. occidentalis* and *F. intonsa* on all three flowers of kidney bean (*P. vulgaris*) cultivar ‘Jiulibai,’ chrysanthemum (*C. morifolium*) cultivar ‘Shenma,’ and rose (*R. rugosa*) cultivar ‘Corolla’ at three sites (Songyang Township Songming County, 25°21′34.55′′N, 102°58′55.19′′E; Xiaojie Township Songming County, 25°20′45.71′′N, 103°05′58.91′′E; and Dounan Township Chenggong district, 24°54′54.06′′N, 102°47’23.60′′E) in Kunming city, Yunnan Province, China. The areas experience a subtropical warm and wet climate. During our sample periods, the average daily rainfall ranged from 4 to 15 mm, average daily relative humidity varied from 65 to 83%, and average daily temperature fluctuated from 16 to 27°C. Because the LC_50_ concentration of imidacloprid in *F. occidentalis* was higher than that of *F. intonsa*, most of *F. intonsa* would be killed if we used this concentration (Hu et al., 2018). Thus, the imidacloprid was applied to thrips population at a LC_50_ concentration of 145 mg.L^–1^ of *F. intonsa* adults every 15 days during the study duration with the control plots receiving clean water (Hu et al., 2018; Zhang et al., 2018). Field sampling extended from 15 March through to 15 August in each year (six samples), with sampling conducted every 30 days from 15 March. At each location, thrips were sampled using the five-point sampling method, with each sampling plot covering an area of approximately 240 m^2^. Four plants of each host (kidney bean, chrysanthemum, and rose) were selected at each sample point, and six flowers from each plant were randomly sampled to count the number of adults of *F. occidentalis* or *F. intonsa*. The thrips on sampled flowers were first collected individually into a plastic tube (50 mL) with a fine brush, labeled, and then transferred into the laboratory for species identification, wherein the male and female adults were counted under stereoscopy (10× to 20×). As with nymphs it is difficult to distinguish species, the collected nymphs were discarded, and were not included in the results of our experiment. In addition, all of the adult thrips samples were stored in the freezer at −80°C before the physiological enzymes were determined, and both species were kept separate (Purcell et al., 1993; Zhang et al., 2017c, 2018).

### Detoxification Enzyme and Antioxidant Enzyme Activity in *F. occidentalis* and *F. intonsa* Under Imidacloprid Stress

Over 200 thrips were collected from the same plant treatment across the six samples per year. The activity of the detoxifying enzymes and antioxidant enzymes was determined at the end of each year’s sampling. Thirty *F. occidentalis* and *F. intonsa* from across all six samples were picked from frozen centrifugal tubes. Anhydrous ethanol was used to extract the enzymes, with four replicates, and 30 thrips for each enzyme in both thrip species.

### Protein Extraction and Antioxidant Enzyme Activity Assay

The total protein content and antioxidant enzyme activity in adults were determined. The protein extraction protocols were carried out according to the manufacturer’s protocol (A045-2, Nanjing Jiancheng Bioengineering Institute, Nanjing, China). Briefly, thirty thrips of each thrip species were placed separately into a 1.5 mL centrifuge tube, and 1,200 L phosphate buffer (pH 7.6, 0.1 mol/L) was added. Specimens were then homogenized using a tissue grinder in ice bath (MY-10 tissue grinder, Shanghai Santeng Instrument Co. Ltd), followed by centrifugation at 10,000 r/min for 15 min (2-16KL type high speed centrifuge, Shanghai Santeng Instrument Co. Ltd). The supernatant was taken as the crude enzyme, and then stored in a refrigerator at 4°C until they were processed within 24 h (Huang et al., 2006). To determine the protein content of the enzyme, the modified coomassie brilliant blue G-250 staining method was used (Bradford, 1976). In brief, bovine serum albumin was used to establish the standard curve of protein in the first step. Two milliliters of 0.01% coomassie brilliant blue G-250 staining solution was mixed thoroughly with 200 μL prepared crude enzyme. After 10 min incubation, the absorbance of 595 nm wavelengths was measured on a spectrophotometer. The concentration of obtained enzyme protein content was calculated using the measured OD value and protein standard curve (mg/L).

The activities of the detoxifying enzymes (CarE, AchE, and MFO) were assayed by using commercially available assay kits (A133-1-1, A024-1-1, H452, Nanjing Jiancheng Bioengineering Institute, Nanjing, China) following the manufacturer’s protocols.

To determine the activity of CarE, 10 μL of stoste detoxification enzyme, 300 μmol/L α - naphthalene acetate 400 μL, 30 μL of 10^–5^mol/L physostigmine, and 560 μL of 0.1 mol/L pH 7.6 phosphoric acid buffer were thoroughly mixed in a centrifuge tube by homogenizer (Vortex-2 type, Shanghai Huxi Instrument Co. Ltd.). After incubation in the water bath for 37 min at 37°C, 100 μL stain solution (5% sodium twelve alkyl sulfate and fast blue B mixed with volume ratio 5:2) was then added, and maintained at room temperature for 10 min. The absorbance of 600 nm wavelengths was measured by a spectrophotometer and the CarE activity was calculated according to the yield of the product (Asperen, 1962).

For determination of AchE activity, 10 μL of detoxification enzyme, 100 μL of 5 × 10^–4^mol/L acetylthiocholine iodide, and 0.9 mL of 0.1 mol/L pH 7.5 phosphoric acid buffers were thoroughly mixed in a centrifuge tube, followed by incubation at 37°C for 20 min in a water bath. Then 1 mL DTNB- phosphoric acid alcohol chromogenic agent was added, and maintained at room temperature for 10 min, then the absorbance of 412 nm wavelengths was measured using a spectrophotometer (UV-1800 spectrophotometer, Shanghai Santeng Instrument Co. Ltd.) and CarE activity calculated according to the yield of the product (Gao, 1987).

Determination of the activity of MFO was based on the method of Liu et al. (2011) with slight modifications. Ten microliter of detoxification enzyme and 50 μL of paranitroanisole (1.0 m mol/L) was mixed in a centrifuge tube and shook well. After a water bath for 5 min under 37°C, 50 μL of reduced coenzyme II (1.0 m mol/L) was added, then the absorbance of 405 nm wavelengths was measured quickly with a spectrophotometer and the MFO activity calculated according to the yield of the product.

CarE, AchE, and MFO was determined spectrophotometrically at 600 nm, 412 nm, and 405 nm, respectively, by measuring the decrease of H_2_O_2_ due to H_2_O_2_ decomposition. One unit of CarE, AchE, and MFO activities were defined as the amount that decomposes 1 μmol of H_2_O_2_ per second per mg protein (U mg^–1^ protein) (Jia et al., 2011; Chen et al., 2018).

### Protein Extraction and Antioxidant Enzymes Assay

Thirty adult thrips were transferred into 2 mL centrifuge tubes. Then 800 μL of 0.4% normal saline was added and homogenized in an ice bath with a tissue grinder. After centrifugation at 2,500 r/min for 10 min, the supernatant was prepared as a coarse enzyme solution. The prepared enzyme solution was stored in the refrigerator at 4°C and used within 24 h. Determination of the protein content of the enzyme solution was the same as coomassie brilliant blue G-250 staining described above.

The antioxidant enzymes (SOD, CAT, and POD) were examined using commercially available assay kits (A001-1-1, A007-1-1, A084-1, Nanjing Jiancheng Bioengineering Institute, Nanjing, China) following the manufacturer’s protocols, and according to the method of Shi et al. (2013). The specific calculation formulas of enzyme activity were as follows:

$$\mathrm{Total}\,\mathrm{SOD}\,\mathrm{activity}\,\left(\text{U}/\mathrm{mg}\,\mathrm{prot}\right)\;=\frac{\text{Blank}\,\text{OD}\,\text{value}-\text{measured}\,\text{OD}\,\text{value}}{\text{Blank}\,\text{OD}\,\text{value}}÷50\%$$

$$\;\times \frac{\mathrm{Total}\,\mathrm{volume}\,\mathrm{of}\,\mathrm{reactive}\,\mathrm{liquid}}{\mathrm{Sampling}\,\mathrm{amount}\,\left(\text{mL}\right)}\;÷\mathrm{Concentration}\,\mathrm{of}\,\mathrm{the}\,\mathrm{sample}\,\mathrm{protein}\,\left(\text{mg}\,\text{prot}/\text{mL}\right)$$

  ⁢CAT⁢activity⁢in⁢tissue⁢(U/mg⁢prot) =(Blank⁢OD⁢value-measured⁢OD⁢value)×271

$$\;\times \frac{1}{60\times \text{sampling}\,\text{amount}}\;÷\mathrm{Concentration}\,\mathrm{of}\,\mathrm{the}\,\mathrm{sample}\,\mathrm{protein}\,\left(\text{mg}\,\text{prot}/\text{mL}\right)$$

$$\;\,\mathrm{POD}\,\mathrm{activity}\,\mathrm{in}\,\mathrm{tissue}\,\left(\text{U}/\mathrm{mg}\,\mathrm{prot}\right)\;=\frac{\text{Measured}\,\text{OD}\,\text{value}-\text{blank}\,\text{OD}\,\text{value}}{12\times \mathrm{Chromatic}\,\mathrm{light}\,\mathrm{diameter}\,\left(1\,\text{cm}\right)}\;\times \frac{\mathrm{Total}\,\mathrm{volume}\,\mathrm{of}\,\mathrm{reactive}\,\mathrm{liquid}\,\left(\mathrm{mL}\right)}{\mathrm{Sampling}\,\mathrm{amount}\,\left(\mu \,L\right)}$$

  ÷Concentration⁢of⁢the⁢sample⁢protein⁢(mg⁢prot/mL)  ×1000

Catalase was determined spectrophotometrically at 405 nm by measuring the decrease of H_2_O_2_ due to H_2_O_2_ decomposition. CAT activities were defined as the amount that decomposes 1 μmol of H_2_O_2_ per second per mg protein (U mg^–1^ protein). POD activity was determined at 420 nm by catalyzing the oxidation of a substrate in the presence of H_2_O_2_. One unit of POD activity was defined as the amount that catalyzes 1 μg substrate per minute per mg protein (U mg^–1^ protein) (Jia et al., 2011; Chen et al., 2018).

### Data Analysis

SAS software (version 9.4) was used for statistical analysis (Liu et al., 2017). The three level processing factors were imidacloprid concentration, host plant species, and thrips species. Three-way ANOVA variance analysis was used to analyze the difference between tested data, and the LSD test was used for multiple comparisons among treatments. *T*-test was used to analyze the enzyme activity difference between these two thrip species.

## Results

### Population Counts of Thrips in the Open Fields

**FIGURE 1:**
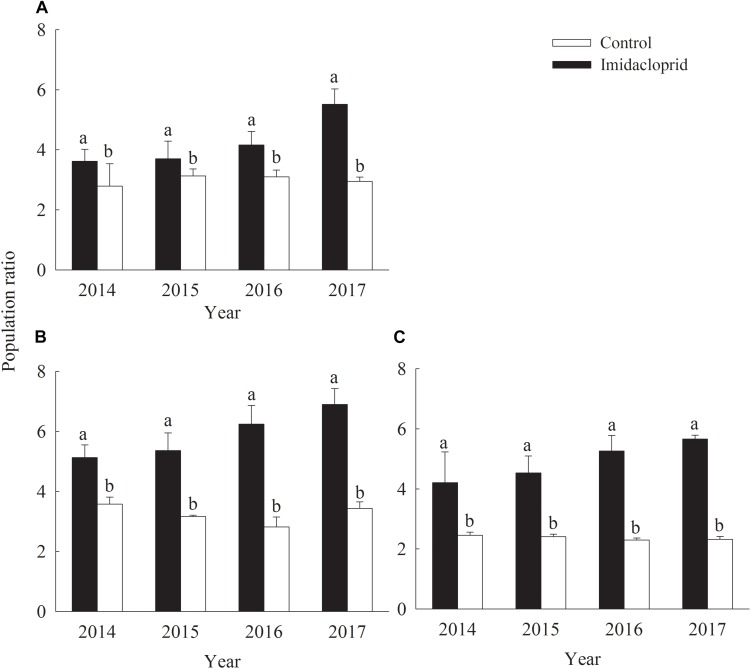
Population ratio of the thrips species *Frankliniella occidentalis* and *F. intonsa* on flowers of three different horticultural plant species in the field. Data in the figure are means (+SE). Different lowercase letters above bars represent significant difference in the population ratio at the same year (LSD, *P* < 0.05). **(A)** Kidney beans. **(B)** Chrysanthemums. **(C)** Roses.

On kidney bean flowers, the mean of each sampled numbers of *F. occidentalis* in imidacloprid treatment (control) from 2014 to 2017 were 42.5 (122.8), 43.4 (119.2), 38.5 (95.3), and 37.6 (99.2), whereas the numbers of *F. intonsa* in imidacloprid treatment (control) were 9.2 (33.9), 11.7 (38.1), 10.2 (30.8), and 10.8 (33.7), respectively (Table 1). The population ratio of *F. occidentalis* and *F. intonsa* increased both in imidacloprid treatment and in control (Figure 1A).

**TABLE 1:** Mean (±SE) population counts of the thrips species *Frankliniella occidentalis* and *F. intonsa* on three different flowers.

Plant species	Thrip species	Year	2014	2015	2016	2017
Kidney bean	*F. occidentalis*	Imidacloprid treatment	42.5 ± 2.4b	43.4 ± 4.8b	38.5 ± 3.1b	37.6 ± 3.9b
		Control	122.8 ± 9.9a	119.2 ± 13.0a	95.3 ± 12.5a	99.2 ± 8.6a
	*F. intonsa*	Imidacloprid treatment	9.2 ± 0.5c	11.7 ± 0.6c	10.2 ± 1.5c	10.8 ± 1.1c
		Control	33.9 ± 2.9b	38.1 ± 2.6b	30.8 ± 2.0bc	33.7 ± 2.6b
Chrysanthemum	*F. occidentalis*	Imidacloprid treatment	112.2 ± 12.6b	110.1 ± 6.4b	114.3 ± 3.7b	120.4 ± 12.6b
		Control	267.9 ± 22.0a	271.7 ± 32.7a	281.3 ± 10.5a	273.1 ± 6.6a
	*F. intonsa*	Imidacloprid treatment	21.9 ± 1.1c	20.6 ± 1.1c	18.3 ± 2.5d	17.4 ± 1.9d
		Control	94.9 ± 16.1b	92.2 ± 3.6b	92.3 ± 2.8c	79.7 ± 6.6c
Rose	*F. occidentalis*	Imidacloprid treatment	184.4 ± 12.9b	166.9 ± 20.1b	149.2 ± 10.0b	156.7 ± 7.7b
		Control	343.9 ± 10.2a	348.8 ± 12.1a	300.0 ± 4.3a	297.3 ± 10.1a
	*F. intonsa*	Imidacloprid treatment	49.7 ± 2.0d	42.9 ± 3.8c	39.2 ± 2.2d	36.2 ± 2.6d
		Control	138.6 ± 10.1c	146.2 ± 8.9b	127.7 ± 5.2c	128.3 ± 10.5c

*Different lowercase letters in the figure indicate significant differences in population density of thrips on the same host plant (LSD, *P* < 0.05).*

In the fields, on chrysanthemum flowers, the total numbers of *F. occidentalis* in imidacloprid treatment (control) from 2014 to 2017 were 112.2 (267.9), 110.1 (271.7), 114.3 (281.3), and 120.4 (273.1), whereas the numbers of *F. intonsa* in imidacloprid treatment (control) were 21.9 (94.9), 20.6 (92.2), 18.3 (92.3), and 17.4 (79.7), respectively (Table 1). The population ratio of both *F. occidentalis* and *F. intonsa* increased for both imidacloprid and the control treatments (Figure 1B).

In the fields, on rose flowers, more *F. occidentalis* were observed than *F. intonsa*. The total number of *F. occidentalis* in the imidacloprid treatment (control) from 2014 to 2017 were 184.4 (343.9), 166.9 (348.8), 149.2 (300.0), and 156.7 (297.3), respectively, whereas the numbers of *F. intonsa* in imidacloprid treatment (control) were 49.7 (138.6), 42.9 (146.2), 39.2 (127.7), and 36.2 (128.3), respectively (Table 1).

In terms of population ratio of these three host plants, field sampling data showed that the ratio of the population of both *F. occidentalis* and *F. intonsa* was significantly higher in the imidacloprid treatment than that in control from 2014 to 2017 on kidney bean (*F*_3_,_8_ = 30.81–84.41, *P* < 0.0001), chrysanthemum (*F*_3_,_8_ = 40.50–359.11, *P* < 0.0001), and rose (*F*_3_,_8_ = 89.67–313.28, *P* < 0.0001) flowers (Figure 1 and Table 1).

### Detoxification Enzyme Activity in *F. occidentalis* and *F. intonsa* Under Imidacloprid Stress

#### Changes in the Activity of Carboxylesterase

**FIGURE 2:**
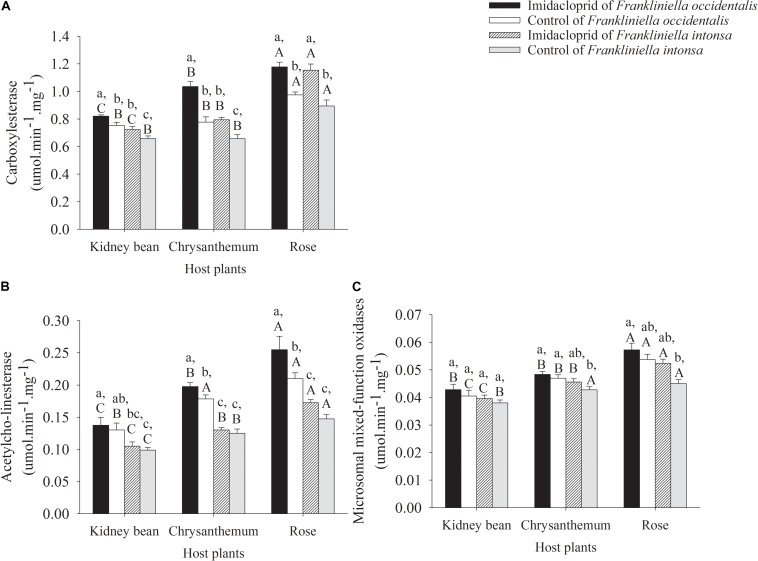
Detoxification enzyme activity in adults thrips under imidacloprid and control treatment on kidney beans, chrysanthemums, and roses. Data in the figure are means (+SE). Different lowercase letters above bars represent significant difference in the detoxification enzyme activities on same host plants between *Frankliniella occidentalis* and *F. intonsa* at the different imidacloprid concentration (LSD, *P* < 0.05), while different uppercase letters above bars indicate significant different in the detoxification enzyme activities for the same thrips species at three different host plants at the same imidacloprid concentration (LSD, *P* < 0.05). The same for Figure 3. **(A)** Carboxylesterase. **(B)** Acetylcho-linesterase. **(C)** Microsomal mixed-function oxidases.

The effects of imidacloprid on CarE activity in adult *F. occidentalis* and *F. intonsa* on three different host plants were shown in Figure 2A. The activity of CarE increased both in these two thrips species subject to imidacloprid exposure compared with the control. The activity of CarE in *F. occidentalis* collected from kidney bean, chrysanthemum, and rose treated with imidacloprid increased by 9.1% (*t* = 2.63, *df* = 6, *P* = 0.0393), 33.2% (*t* = 4.96, *df* = 6, *P* = 0.0025), and 20.8% (*t* = 5.04, *df* = 6, *P* = 0.0024), compared to the corresponding controls, respectively (Figure 2A). The activity of CarE in *F. intonsa* exposed to imidacloprid on kidney bean, chrysanthemum, and rose increased by 10.0% (*t* = 2.19, *df* = 6, *P* = 0.0707), 20.8% (*t* = 4.13, *df* = 6, *P* = 0.0061), and 30.0% (*t* = 4.19, *df* = 6, *P* = 0.0057), compared to thrips on the corresponding control plants, respectively (Figure 2A). For *F. occidentalis* exposed to imidacloprid, there was a significant host plant effect on CarE activity with the highest for specimens recovered from roses, followed by chrysanthemum, then kidney beans (*F*_2_,_9_ = 38.14, *P* < 0.0001). A similar trend was found with CarE activity of *F. intonsa* on three plant flowers under imidacloprid stress, with highest CarE activity found on the rose population, and kidney with the lowest one (*F*_2_,_9_ = 58.85, *P* < 0.0001). In the control, CarE activity of the *F. intonsa* on rose was significantly higher than that of on chrysanthemum and kidney bean (*F*_2_,_9_ = 18.17, *P* = 0.0007). However, there were no differences of the CarE activity between chrysanthemum and kidney bean. Furthermore, on the same host plant, CarE activity in *F. occidentalis* was higher than that of *F. intonsa* both in imidacloprid stress treatments (kidney bean: *F*_1_,_6_ = 16.40, *P* = 0.0067; chrysanthemum: *F*_1_,_6_ = 37.70, *P* = 0.0009; rose: *F*_1_,_6_ = 0.17, *P* = 0.6944) and in control (kidney bean: *F*_1_,_6_ = 7.18, *P* = 0.0439; chrysanthemum: *F*_1_,_6_ = 6.36, *P* = 0.0451; rose: *F*_1_,_6_ = 2.76, *P* = 0.1476). On the kidney bean, *F. occidentalis* held higher CarE activity than that of *F. intonsa* both on kidney bean (*F*_3_,_12_ = 11.65, *P* = 0.0007) and on chrysanthemum (*F*_3_,_12_ = 26.39, *P* < 0.0001), the activity of CarE in imidacloprid treatment *F. occidentalis* and *F. intonsa* were higher than that in control, and there was no difference of CarE activity between *F. occidentalis* and *F. intonsa* on rose (*F*_3_,_12_ = 13.90, *P* = 0.0003) (Figure 2A). The change in the activity of CarE in the *F. occidentalis* on chrysanthemum was biggest (in imidacloprid treatment: *F*_2_,_9_ = 38.14, *P* < 0.0001; in control: *F*_2_,_9_ = 18.04, *P* = 0.0007). However, the increase in activity of CarE in the *F. intonsa* on rose was greatest (in imidacloprid treatment: *F*_2_,_9_ = 58.85, *P* < 0.0001; in control: *F*_2_,_9_ = 18.17, *P* = 0.0007). The CarE activity increased least in both of the thrip species on kidney beans (*F. occidentalis*: *F*_2_,_9_ = 4.34, *P* = 0.0480; *F. intonsa*: *F*_2_,_9_ = 58.93, *P* < 0.0001), compared with the other two host plants (Figure 2A).

#### Changes in the Activity of Acetylcho-Linesterase

The activities of AchE were increased in both these two thrips, but the degree of increase was different under imidacloprid exposure. Compared with the control, the activity of AchE in *F. occidentalis* on kidney bean, chrysanthemum, and rose under imidacloprid stress was increased by 5.7% (*t* = 0.45, *df* = 6, *P* = 0.6684), 10.8% (*t* = 12.14, *df* = 6, *P* = 0.0062), and 21.4% (*t* = 22.00, *df* = 6, *P* < 0.0001), respectively (Figure 2B). Compared with the control, the activity of AchE in *F. intonsa* on kidney bean, chrysanthemum, and rose under imidacloprid stress was increased by 6.3% (*t* = 0.81, *df* = 6, *P* = 0.4502), 4.0% (*t* = 0.65, *df* = 6, *P* = 0.5370), and 17.0% (*t* = 3.16, *df* = 6, *P* = 0.0195), respectively (Figure 2B). Regarding the activity of AchE, the results showed similar trends both in *F. occidentalis* and *F. intonsa* in control and under the imidacloprid stress, with the highest on rose among the three host plants. The activities of AchE both in two thrips on three host plants were as follows: rose > chrysanthemum > kidney bean. In addition, the activities of AchE in these two thrips on the same host plant were *F. occidentalis* > *F. intonsa* (kidney bean: *F*_1_,_6_ = 5.42, *P* = 0.0588; chrysanthemum: *F*_1_,_6_ = 81.00, *P* = 0.0001; rose: *F*_1_,_6_ = 15.20, *P* = 0.0080; in imidacloprid treatments, kidney bean: *F*_1_,_6_ = 7.24, *P* = 0.0360; chrysanthemum: *F*_1_,_6_ = 34.14, *P* = 0.0011; rose: *F*_1_,_6_ = 31.78, *P* = 0.0013; in control) (Figure 2B). *F. occidentalis* had higher AchE activity than that of *F. intonsa* on kidney bean (*F*_3_,_12_ = 4.30, *P* = 0.0281), chrysanthemum (*F*_3_,_12_ = 36.78, *P* < 0.0001), and on rose (*F*_3_,_12_ = 15.37, *P* = 0.0002) (Figure 2B).

#### Changes in the Activity of Microsomal Mixed-Function Oxidases

Compared with the control, the activity of MFO in *F. occidentalis* on kidney beans, chrysanthemum, and roses increased by 5.8% (*t* = 0.82, *df* = 6, *P* = 0.4452), 2.8% (*t* = 0.78, *df* = 6, *P* = 0.4633), and 6.5% (*t* = 1.13, *df* = 6, *P* = 0.3023) treated with imidacloprid, respectively (Figure 2C); whereas the activity of MFO in *F. intonsa* on kidney beans, chrysanthemum, and roses increased by 4.1% (*t* = 0.96, *df* = 6, *P* = 0.3730), 6.7% (*t* = 1.77, *df* = 6, *P* = 0.1275), and 16.4% (*t* = 3.45, *df* = 6, *P* = 0.0137) under imidacloprid stress, respectively (Figure 2C). The activity of MFO in *F. occidentalis* on rose was highest both in control (*F*_2_,_9_ = 13.65, *P* = 0.0019) and in imidacloprid stress (*F*_2_,_9_ = 14.35, *P* = 0.0016), followed by chrysanthemum. Kidney bean held the lowest activity of MFO. However, there was no significant difference in the activity of MFO on chrysanthemum and kidney beans under imidacloprid stress. For *F. intonsa*, the activities of MFO on rose and chrysanthemum was significantly higher than that on kidney beans in control (*F*_2_,_9_ = 8.40, *P* = 0.0088), whereas, the activity of MFO on rose was the highest among the three host plants, but there was no significantly difference between chrysanthemum and kidney beans under imidacloprid stress (*F*_2_,_9_ = 23.32, *P* = 0.0003). Briefly, the activities of MFO in two species of thrips on these three host plants were as follow: rose > chrysanthemum > kidney bean, and the activities of MFO in two thrips on the same host plant were as fellow: *F. occidentalis* > *F. intonsa* both in control and in treatment. Rose held the widest increasing range on the activity of MFO in these two thrips species both in control and in treatment (Figure 2C). There were no significant differences of MFO activity between *F. occidentalis* and *F. intonsa* on kidney beans (*F*_3_,_12_ = 1.50, *P* = 0.2640); the activities of MFO in imidacloprid treatment was higher than that in control both on chrysanthemum (*F*_3_,_12_ = 4.21, *P* = 0.0298) and on rose (*F*_3_,_12_ = 7.50, *P* = 0.0044) in these two thrips species (Figure 2C).

#### The Three-Way ANOVA Analysis of Detoxifying Enzymes

The results of three-way ANOVA analysis showed that the F values of activity correction models (Corrected Model) of the CarE, AchE, and MFO of *F. occidentalis* and *F. intonsa* were 35.9, 25.0, and 14.0, respectively. CarE activity was significantly affected by the species of host plants, imidacloprid stress, thrips species, host plant species × imidacloprid stress, and host plant species × thrips species (*F*_1_,_32_ = 116.82, *P* < 0.0001; *F*_1_,_32_ = 90.72, *P* < 0.0001; *F*_1_,_32_ = 39.86, *P* < 0.0001; *F*_3_,_32_ = 8.30, *P* = 0.0011; *F*_3_,_32_ = 4.76, *P* = 0.0147, respectively). The activity of AchE was also significantly influenced by the host plant species, imidacloprid stress, thrips species, and interaction between host plant species × thrips species (*F*_1_,_32_ = 71.16, *P* < 0.0001; *F*_1_,_32_ = 11.21, *P* = 0.0019; *F*_1_,_32_ = 104.47, *P* < 0.0001; *F*_3_,_32_ = 5.04, *P* = 0.0117, respectively). The activity of MFO was significantly affected by the host plant species, imidacloprid stress, and thrips species (*F*_1_,_32_ = 55.32, *P* < 0.0001; *F*_1_,_32_ = 11.75, *P* = 0.0015; *F*_1_,_32_ = 22.8, *P* < 0.0001, respectively). There were no significant differences in the activities of these three detoxifying enzymes of thrips species × imidacloprid stress and host plant species × thrips species × imidacloprid stress (Table 2).

**TABLE 2:** Three-way ANOVA analysis on the activities of detoxifying enzymes.

Factors	Carboxylesterase activity			Acetylcho-linesterase activity			Microsomal mixed-function oxidases activity		
	*df*	*F*	*P*	*df*	*F*	*P*	*df*	*F*	*P*
Host plant species	2	116.82	<0.0001	2	71.16	<0.0001	2	55.32	<0.0001
Imidacloprid stress	1	90.72	<0.0001	1	11.21	0.0019	1	11.75	0.0015
Thrip species	1	39.86	<0.0001	1	104.47	<0.0001	1	22.8	<0.0001
Host plant species × imidacloprid stress	2	8.30	0.0011	2	2.59	0.0888	2	0.7	0.4084
Host plant species × thrip species	2	4.76	0.0147	2	5.04	0.0117	2	1.77	0.1854
Thrip species × imidacloprid stress	1	0.42	0.5233	1	1.21	0.2796	1	0.7	0.4084
Host plant species × thrip species × imidacloprid stress	2	2.28	0.117	2	0.27	0.7646	2	0.53	0.5943
									

### Determining Activity of Antioxidant Enzymes in Adult Thrips

#### Changes in the Activity of Superoxide Dismutase

**FIGURE 3:**
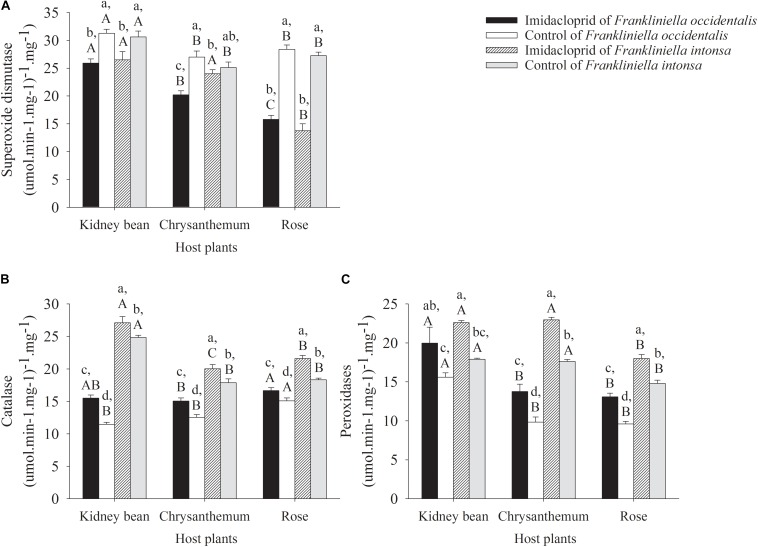
Antioxidant enzyme activity in adults thrips under imidacloprid and control treatment on kidney beans, chrysanthemums and roses. **(A)** Superoxide dismutase. **(B)** Catalase. **(C)** Peroxidases.

The activity of SOD in adult *F. occidentalis* and *F. intonsa* on rose, chrysanthemum, and kidney bean decreased under imidacloprid stress. Compared with the control, the activities of SOD in *F. occidentalis* reduced in kidney bean by 17.2% (*t* = −5.02, *df* = 6, *P* = 0.0024), chrysanthemum by 25.2% (*t* = −5.24, *df* = 6, *P* = 0.0019), and rose by 44.3% (*t* = −11.84, *df* = 6, *P* < 0.0001), respectively (Figure 3A). Compared with the control, the activities of SOD in *F. intonsa* reduced in kidney bean by 13.5% (*t* = −2.29, *df* = 6, *P* = 0.0623), chrysanthemum by 4.5% (*t* = −0.92, *df* = 6, *P* = 0.3951), and rose by 49.5% (*t* = −9.65, *df* = 6, *P* < 0.0001), respectively. For *F. occidentalis* collected from the control plots, thrips collected from kidney beans showed the highest activity of SOD compared to that recorded from chrysanthemum and rose (*F*_2_,_9_ = 6.08, *P* = 0.0213). While under imidacloprid treatment, samples collected from kidney beans held the highest and roses held the lowest activity of SOD (*F*_2_,_9_ = 47.52, *P* < 0.0001). For *F. intonsa*, collected from the control plots, kidney bean population held the highest activity of SOD of the three host plants (*F*_2_,_9_ = 9.40, *P* = 0.0062). However, of *F. intonsa* collected from the imidacloprid treatments, kidney beans and chrysanthemum had significantly higher activity of SOD than that of rose (*F*_2_,_9_ = 32.06, *P* < 0.0001). For both species, the activity of SOD on rose descended most sharply under imidacloprid treatment, compared with control (Figure 3A). Compared to the control, imidacloprid treatment significantly decreased the SOD activities in these two thrip species both on kidney beans (*F*_3_,_12_ = 6.94, *P* = 0.0058) and on rose (*F*_3_,_12_ = 74.63, *P* < 0.0001). There was no significant differences between *F. occidentalis* and *F. intonsa* both in control and in imidacloprid treatment on these two host plants species; whereas the activities of SOD in imidacloprid treatment *F. intonsa* was higher than that of *F. occidentalis* on chrysanthemum (*F*_3_,_12_ = 10.32, *P* = 0.0012) (Figure 3A).

#### Changes in the Activity of Catalase

Catalase activity in adult *F. occidentalis* and *F. intonsa* on rose, chrysanthemum, and kidney beans increased with imidacloprid treatment. CAT in *F. occidentalis* on kidney beans enhanced by 35.3% (*t* = 6.90, *df* = 6, *P* = 0.0005), chrysanthemum enhanced by 19.8% (*t* = 3.88, *df* = 6, *P* = 0.0082), and rose enhanced by 10.5% (*t* = 2.51, *df* = 6, *P* = 0.0459), respectively. CAT activity on these three host plants of *F. intonsa* declined in imidacloprid treatment compared with the control, with kidney beans decreasing 9.1% (*t* = 5.76, *df* = 6, *P* = 0.0012), chrysanthemum declining 12.0% (*t* = 2.39, *df* = 6, *P* = 0.0539), and rose reducing 17.8% (*t* = 2.58, *df* = 6, *P* = 0.0401), respectively. The rose population of *F. occidentalis* had the highest CAT activity both in control (*F*_2_,_9_ = 21.13, *P* = 0.0004) and in imidacloprid stress (*F*_2_,_9_ = 6.11, *P* = 0.0339). For *F. intonsa*, the kidney bean population had the highest CAT activity than that of chrysanthemum or rose in control (*F*_2_,_9_ = 85.12, *P* < 0.0001) (Figure 3B). The CAT activities of *F. intonsa* was higher than that of *F. occidentalis* both in control and in imidacloprid treatment on kidney beans (*F*_3_,_12_ = 446.50, *P* < 0.0001), chrysanthemum (*F*_3_,_12_ = 35.09, *P* < 0.0001), and on rose (*F*_3_,_12_ = 43.94, *P* < 0.0001) (Figure 3B).

#### Changes in the Activity of Peroxidases

The activities of POD in adult *F. occidentalis* and *F. intonsa* on roses, chrysanthemums, and kidney beans increased at different levels under imidacloprid stress (Figure 3C). Compared with the control, the activity of POD of *F. occidentalis* on the three different host plants increased under imidacloprid exposure with kidney beans increasing by 28.1% (*t* = 2.05, *df* = 6, *P* = 0.0866), chrysanthemum rising by 39.9% (*t* = 3.44, *df* = 6, *P* = 0.0138), and rose increasing by 36.2% (*t* = 6.12, *df* = 6, *P* = 0.0009), respectively. Compared with the control, the activity of POD of *F. intonsa* on three different host plants decreased under imidacloprid stress, which on kidney bean decreased by 26.3% (*t* = 16.11, *df* = 6, *P* < 0.0001), chrysanthemum reduced by 30.3% (*t* = 13.18, *df* = 6, *P* < 0.0001), and rose declined by 21.7% (*t* = 4.72, *df* = 6, *P* = 0.0033), respectively. For adult *F. occidentalis*, the population on kidney beans had the highest POD activity (*F*_2_,_9_ = 39.92, *P* < 0.0001; *F*_2_,_9_ = 8.10, *P* = 0.0097). However, there were no difference in POD activity between chrysanthemum and rose both in control and imidacloprid treatment. For adult *F. intonsa*, on kidney beans and chrysanthemum, the population held higher POD activity than that on rose in control (*F*_2_,_9_ = 32.14, *P* < 0.0001) and in treatment (*F*_2_,_9_ = 53.01, *P* < 0.0001) (Figure 3C). There were no differences of POD activities between *F. occidentalis* and *F. intonsa* on kidney beans (*F*_3_,_12_ = 7.67, *P* = 0.0040), while the POD activities of *F. intonsa* was higher than that of *F. occidentalis* on chrysanthemum (*F*_3_,_12_ = 85.37, *P* < 0.0001) and on rose (*F*_3_,_12_ = 62.53, *P* < 0.0001) (Figure 3C).

#### The Three-Way ANOVA Analysis of Antioxidant Enzymes

The results of three-way ANOVA showed that the *F*-values of SOD, CAT, and POD activity (Corrected Model) for both species were 33.5, 110.4, and 33.4, respectively. There was a significant difference between imidacloprid exposed and control for each of the three indicator enzymes, and therefore the selected model had statistical significance since the accompanying probability was less than 0.0001. The effects of host plant species, imidacloprid stress, and host plant species × imidacloprid stress on SOD activity were significant (*F*_1_,_32_ = 60.6, *P* < 0.0001; *F*_1_,_32_ = 177.13, *P* < 0.0001; *F*_3_,_32_ = 28.40, *P* < 0.0001, respectively). The host plants species, imidacloprid stress, thrips species, and the host plants species × thrips species have significant effects on the activity of CAT and POD (CAT: *F*_1_,_32_ = 56.09, *P* < 0.0001; *F*_1_,_32_ = 103.16, *P* < 0.0001; *F*_1_,_32_ = 781.43, *P* < 0.0001; *F*_3_,_32_ = 104.04, *P* < 0.0001, respectively; POD: *F*_1_,_32_ = 46.45, *P* < 0.0001; *F*_1_,_32_ = 90.82, *P* < 0.0001; *F*_1_,_32_ = 148.83, *P* < 0.0001; *F*_3_,_32_ = 15.87, *P* < 0.0001, respectively). No significant differences were observed in the activities of three antioxidant enzymes in thrips species × imidacloprid stress and thrips × imidacloprid stress × host plants (Table 3).

**TABLE 3:** Three-way ANOVA analysis on the activities of antioxidant enzymes.

Factors	Superoxide dismutase activity			Catalase activity			Peroxidases activity		
	*df*	*F*	*P*	*df*	*F*	*P*	*df*	*F*	*P*
Host plant species	2	60.60	<0.0001	2	56.09	<0.0001	2	46.45	<0.0001
Imidacloprid stress	1	177.13	<0.0001	1	103.16	<0.0001	1	90.82	<0.0001
Thrip species	1	0.16	0.6925	1	781.43	<0.0001	1	148.83	<0.0001
Host plant species × imidacloprid stress	2	28.40	<0.0001	2	1.03	0.366	2	0.9	0.4149
Host plant species × thrip species	2	1.87	0.169	2	104.04	<0.0001	2	15.87	<0.0001
Thrip species × imidacloprid stress	1	3.35	0.0756	1	0.09	0.7664	1	0.31	0.5793
Host plant species × thrip species × imidacloprid stress	2	3.20	0.0528	2	2.77	0.0507	2	0.32	0.7283

## Discussion

Two important findings can be seen in the present study. First, in the field investigation, the population size of *F. occidentalis* was larger than that of *F. intonsa* on all three horticultural crops with roses supporting the highest population of *F. occidentalis*. Second, in the laboratory, the activity of the detoxifying enzymes CarE, AchE, MFO, and antioxidant enzymes CAT and POD of these two thrips increased after imidacloprid exposure, suggesting that the constant use of imidacloprid for controlling in the invasive species *F. occidentalis* might exert an important impact on increased proportion of population in thrips, leading to population outbreaks in the field, which needs to be further studied.

Investigation of the population dynamics of *F. occidentalis* and *F. intonsa* in our study showed that *F. occidentalis* maintained a higher population density than that of *F. intonsa* on all three host plants. The differences in population on different host plants indicated competition against each other when they held a close ecological niche. Furthermore, native species are often displaced by invasive species when they are in the same ecological environment (Cao et al., 2018; Hu et al., 2018). Invasive species *F. occidentalis* held a stronger competition ability than the native species *F. intonsa*. For instance, *F. occidentalis* has displaced *F. gemina* in Argentina on *Lycopersicon esculentum* (De Piìccolo et al., 2006), and *Thrips hawaiiensis* has been displaced by *F. occidentalis* on *Rosa chinensis* and *Gardenia jasminoides*, Cao et al. (2018). Research also found *Thrips tabaci* was the dominant species on *Brassica oleracea* when no insecticide was used but was displaced by *F. occidentalis* under avermectin and beta-cypermethrin use (Zhao et al., 2017). Thus, insecticides could be an important factor in intensifying the competition between *F. occidentalis* and *T. tabaci* in eastern China (Zhao et al., 2017). In our study, we found that *F. occidentalis* maintained a higher population density than *F. intonsa* in three of the sampled host plants in most cases, especially in imidacloprid-used fields. In a previous study, *F. occidentalis* was more resistant than *F. intonsa*, with females being more tolerant than males for both species under imidacloprid stress (Hu et al., 2018). A higher population ratio of *F. occidentalis* and *F. intonsa* in rose flowers than that on kidney bean flowers indicated that thrips had a host plant preference (Wang et al., 2013). Because outdoor experiments are influenced by seasonal effects and variable environmental factors within seasons, there were no obvious correlations between population dynamics amongst years. Therefore, to objectively evaluate the population performance of both *F. occidentalis* and *F. intonsa* in relation to host plant and insecticide exposure, the population development of thrips needs to be done under laboratory conditions, where environmental factors can be controlled and subtle changes in population growth parameters can also be carefully monitored.

Environmental changes can largely affect the feeding, survival, and reproduction of thrips because of its tiny size, and thrips themselves are extremely sensitive to changes in their surrounding environment. Therefore, the thrips must quickly adapt to some changes in the environment and find more suitable survival or ecological countermeasures. Our results also showed that the activities of various enzymes in these two thrips species changed at different levels under imidacloprid treatment for a long time, although the response of *F. occidentalis* and *F. intonsa* to environmental changes were inconsistent on different host plants.

CarE, AchE, and MFO are the important physiological metabolic detoxification enzymes in insects, playing an important role in decomposing environmental toxins, maintaining normal physiological metabolism, and buffering against exposure (Xu et al., 2006). In our study, the activities of these three detoxifying enzymes increased in both *F. occidentalis* and *F. intonsa on* three host plants under imidacloprid exposure, and the increasing level of the detoxifying enzymes in *F. occidentalis* were much larger than that of *F. intonsa*. The population of these two thrips species in chrysanthemum and rose showed an overall trend of increase. Our findings were consistent with other researcher’s findings. Liu et al. (2017) found that adversity, environmental change, and stress (e.g., increased CO_2_ concentration) increased the activity of these three enzymes in these two thrips species.

The Wang et al. (2016) study showed that the use of high concentration of imidacloprid (>0.010 mg/L) can promote SOD and CarE activity in adult *Ambrostoma quadriimopressum*. Imidacloprid stress had a promoting effect on the activity of CAT, POD, and SOD in *Coloana cinerea* (Sun et al., 2015). Spinosad stress can improve the activities of major detoxifying enzymes and antioxidant enzymes, such as CarE, AchE, SOD, POD, and CAT in four instar larvae of *Malacosoma neustria testacea* (Liu et al., 2012). Imidacloprid can effectively interfere with the detoxification and protection systems of insects and disrupt their normal physiological metabolism, which could have a poisonous effect on insects (Liu et al., 2012). *F. occidentalis* have a stronger capability for resistance to toxicity of imidacloprid than that of *F. intonsa*, and females were stronger than males for both thrips (Zhang et al., 2018). The CAT and POD were increased in *F. occidentalis* and *F. intonsa* under the stress of imidacloprid; the production of POD and CAT in thrips can be activated to adapt to the invasion of exogenous toxins, which then degrades toxicity and protects them from imidacloprid harm (Liu et al., 2018), meanwhile, the reason why SOD activities were decreased needs further study. Longevity of male and female adults, average fecundity, the net reproductive rate (*R*_0_), the intrinsic rate of increase (*r*_m_), and the rate of growth (λ) on *F. occidentalis* were significantly higher than that on *F. intonsa* under imidacloprid stress (Hu et al., 2018). In this study, we found that the enzymes changes are bigger in *F. occidentalis* than that of *F. intonsa* under imidacloprid stress in most cases, and the population of *F. occidentalis* was larger than that of *F. intonsa* under imidacloprid stress. Therefore, it could be elucidated that imidacloprid stress could be an important environmental factor in competitive substitution in thrips *F. occidentalis* to *F. intonsa.*

Our results showed that different host plants could significantly affect the activity of detoxifying enzymes and antioxidant enzymes in thrips. The activities of detoxifying enzymes and antioxidant enzymes in *F. occidentalis* and *F. intonsa* in rose changed more significantly than that in kidney beans. Liu et al. (2011) studied the effects of different rice varieties (TN1, IR42, RHT) and *japonica* rice varieties Wu Yu Jing-3, and the common weeds barnyard grass in rice fields on detoxification and antioxidant enzyme activities in three Homoptera pests: Planthoppidae *Nilaparvata lugens*, *S. furcifera*, and *Laodelphax striatellus*. They found that different host plants had different effects on the activities of CarE, SOD, and POD in these three species of plant hoppers, and hosts had the most significant influence on the *L. striatellus* and the least influence on the *Nilaparvata lugens*. Yin et al. (2012) determined the sequential variation in the activity of the detoxification enzyme carboxylic acid esterase, AchE and antioxidant enzyme SOD, CAT, POD activity of larvae *Loxostege sticticalis* in five different host plants comprising *Amaranthus retroflexus*, *Glycine max*, *Helianthus annuus*, *Zea mays*, and *Solanum tuberosum*. The results showed that the activities of detoxifying enzymes and antioxidant enzymes in larval midgut were significantly lower for larvae feeding on the host plant *Amaranthus retroflexus* compared to cohorts feeding on the four remaining non-host plants. In another study, the activity of SOD of *Bemisia tabaci* biotype B in *Solanum melongena* was 2.64-fold than that in *Lycopersicon esculentum*. The activity of POD in *B. tabaci* biotype B on *S. melongena* was 3.13-fold than that in *Abutilon avicennae*. *S. melongena* supported the highest CarE activity, followed by the *A. avicennae*, *S. tuberosum*, and the lowest was *Cucumis sativus* for *B. tabaci* biotype B, thus the enzyme activity was dependent on the plant species on which the insect was feeding (Zhou et al., 2011). Insects can adapt to environmental changes by regulating and changing their CarE activity, which could reduce the harm they experience from unfavorable environments and adversity exposure (Terriere and Yu, 1974). In our experiments, the activities of MFO in populations of *F. occidentalis* and *F. intonsa* were significantly different in kidney beans and chrysanthemum under imidacloprid exposure. There was no significant difference between control and treatment of the MFO activities in *F. occidentalis* feeding on roses, while the activity of MFO in *F. intonsa* increased significantly after exposure to imidacloprid in rose. The changes of enzyme activity were sometimes inconsistent with the level of enzyme accumulation in the neurotransmitter of *F. occidentalis* and *F. intonsa* (Gatehouse et al., 1992). This could be caused by the difference of nutrient contents and secondary substances in plant tissues. For instance, Qian et al. (2015) found that elevated CO_2_ concentration can change the nutritional ingredients of kidney bean leaves, which affected the growth, development, and reproduction of *F. occidentalis*. The SOD activity of *Aphis gossypii* (Ge et al., 2010) under low temperature exposure and of *F. occidentalis* (Shi et al., 2013) under high CO_2_ concentration also showed similar results. Three kinds of oxidase in thrips on different hosts could have different response patterns to imidacloprid exposure. This might be related to the specific circumstances of environmental changes and the biological characteristics of insects themselves. In this study, it was found that the activities of SOD, CAT, and POD in *F. occidentalis* and *F. intonsa* were at different levels in these three different host plants under imidacloprid exposure, and the difference among different host plants was also significant. However, in our study, the enzyme activities of different generations were not compared under the different population residence time and exposure time.

Changes in the activity of detoxification enzymes in these two thrips species might result from the increase in food intake by the *F. occidentalis* and *F. intonsa* in order to adapt to the exposure of the insecticide. As such, the enzyme activity of these two thrips also increased significantly even in the unsuitable host plant kidney bean. However, due to the different species and content of secondary metabolite in different host plants, the content of detoxification enzymes produced by insects under imidacloprid and other exposures was different, which could result in different adaptabilities of insects to different host plants (Yin et al., 2004). The activity of CarE in these two kinds of thrips on kidney beans increased at a lower level, indicating that different host plants could only stimulate some detoxifying enzymes in these two kinds of thrips (Yin et al., 2012). This is the mechanism used by insects to adapt to the changes in host plant’s metabolic pathways during the adaptive evolution of insects. It could also help insects to detoxify and save energy (Yin et al., 2012).

The interaction analysis of host plants, imidacloprid exposure, and thrips species showed that imidacloprid exposure and thrips species were the main factors resulting in the change of the three antioxidant enzymes, but the host plants were the main factors that affected the activity of SOD and CAT in thrips. Invasive species *F. occidentalis* has the greater physiological tolerance and wider ecological adaptability compared with the native thrips such as *F. intonsa* (Hertling and Lubke, 2000; Baez et al., 2011). Our study showed that the change of POD activities were different in *F. occidentalis* and *F. intonsa* on kidney beans, chrysanthemum, and roses under imidacloprid exposure conditions. It was speculated that the difference of environmental adaptability of *F. occidentalis* and *F. intonsa* under imidacloprid exposure would lead to a change in enzyme activity, and this would play an important role in the process of competition and substitution between these two thrips. In our study, there was no significant interaction among host species, imidacloprid exposure, and thrips species on the activities of detoxifying enzymes and antioxidant enzymes in thrips, which is mainly due to many factors that affect the enzyme activity in thrips. The synthesis of detoxifying enzymes and antioxidant enzymes is regulated by the nerves of thrips, and the process is complicated. In brief, our present results provide a more detailed explanation for the outbreak of invasive pest insects *F. occidentalis*, and the different physiological response of thrips on different host plants may indicate different host-plant resistances to different thrips species. We need to use more effective and practical approaches for protecting crops from *F. occidentalis* and mitigating the chemical pressure on horticultural crops.

## Data Availability Statement

The datasets generated for this study are available on request to the corresponding author.

## Author Contributions

XZ, GC, and ZL conceived and designed the research. XZ, CH, HX, and ZC conducted the experiments. XZ analyzed the data. XZ and RL wrote the manuscript. All authors read, commented on, and approved the manuscript.

## Conflict of Interest

ZC was employed by Kunming Hongzhihua Horticulture Co., Ltd. of China. The remaining authors declare that the research was conducted in the absence of any commercial or financial relationships that could be construed as a potential conflict of interest.
